# A comparison of excess deaths by UK country and region during the first year of the COVID-19 pandemic

**DOI:** 10.1093/eurpub/ckad144

**Published:** 2023-10-19

**Authors:** Neil A Hopper, Annie Campbell, Cath Roberts, Julie Ramsay, Jos IJpelaar, Myer Glickman, Vahé Nafilyan, Nazrul Islam

**Affiliations:** Data Analysis of Social Care and Health, Office for National Statistics, Newport, UK; Health, Social Services and Population Statistics, Welsh Government, Cardiff, UK; Health, Social Services and Population Statistics, Welsh Government, Cardiff, UK; Demographic Statistics, National Records Scotland, Edinburgh, UK; Administrative Research Unit, Northern Ireland Statistics and Research Agency, Belfast, UK; Epidemiology and Global Health Analysis, Office for National Statistics, Newport, UK; Data Analysis of Social Care and Health, Office for National Statistics, Newport, UK; Data Analysis of Social Care and Health, Office for National Statistics, Newport, UK; Faculty of Medicine, University of Southampton, Southampton, UK

## Abstract

We compare the impact of the first two waves of the COVID-19 pandemic on risk of age-standardized mortality by sex, UK country, and English region. Each wave is defined as lasting 26 weeks and are consecutive beginning in 2020 week 11. The expected rate is estimated from 2015 to 2019 mean and the projected mortality trend from the same period are used to estimate excess mortality. By both measures, excess mortality was highest and lowest in regions of England, London and the South-West, respectively. Excess mortality was consistently higher for males than females.

## Introduction

The impact of the COVID-19 pandemic upon all-cause mortality in the UK is assessed through excess deaths based on weekly death registrations for 52 weeks starting from 2020 week 11, which saw the first registered COVID-19 death. All-cause mortality captures indirect and direct deaths that may have been caused by COVID-19 but not attributed to it.^1–3^ Analysing regional differences in mortality helps inform local clinical and public health policy of relative healthcare need. Our aim is to estimate regional inequalities in mortality between Northern Ireland, Scotland, Wales, and regions of England across the first two waves of the COVID-19 pandemic.

## Methods

Weekly deaths by quinary age, sex, and week of registration were obtained from constituent countries of the UK. Registration weeks run from Saturday to Friday in the UK except in Scotland where they run Monday to Sunday.^4^ Population estimates are extrapolated for each week from mid-year estimates to 2020,^5^ with values from July 2020 carried forward from the 2020 Mid-Year Estimates. The analysis is split into two periods of equal durations, with week 36, which in 2020 had the lowest observed overall death registrations,^6^ defining the end of the first period. Wave one from week 11–36 and wave two from week 37 to week 9 in the following year. There are 5 years of historical data, beginning from week 11 in 2015 and ending in 2020 week 9, from which expected deaths are derived. Wave one contains 26 weeks in all years. Wave two contains 26 weeks in 2015 and 2020 (these are 53-week years), but 25 weeks in 2016–2019. Deaths in the historic wave two periods of 25 weeks are weighted 26/25 to adjust.

The analysis is sensitive to the method used to estimate expected values.^7^ Two methods are used here. In line with previous National Statistics mortality publications,^4^^,^^6^ a simple 5-year average expected crude mortality rate (CMR-sm) was calculated by region, country, and sex across each wave period. Since previous studies showed that a 5-year average tends to over-estimate expected mortality, and consequently underestimate excess mortality, we also used a previously published methodology to estimate counterfactual (projected) deaths based on historic trend in mortality.^8^ To maintain statistical stability, we used 10-year age groups (0–9, 10–19, through 80–89 and 90 plus). Excess deaths were estimated as the difference between estimated expected deaths (if the pandemic had not occurred) from the observed deaths. To facilitate comparison across regions and waves, age-standardized mortality rates [ASMR-sm (simple mean) and ASMR-proj (projected)] were calculated by weighting to the 2013 European standard population.

## Results

**Figure 1.**
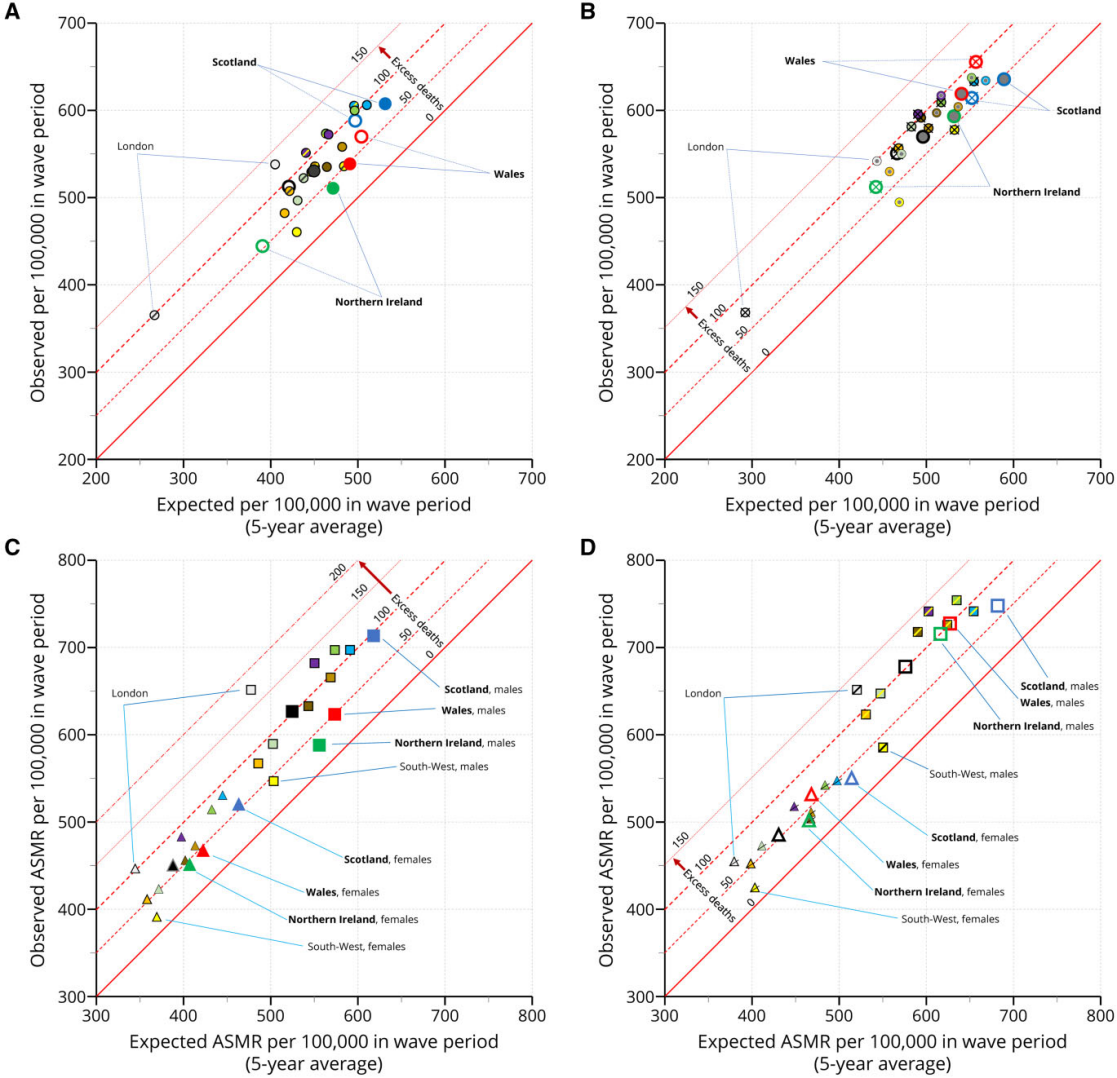

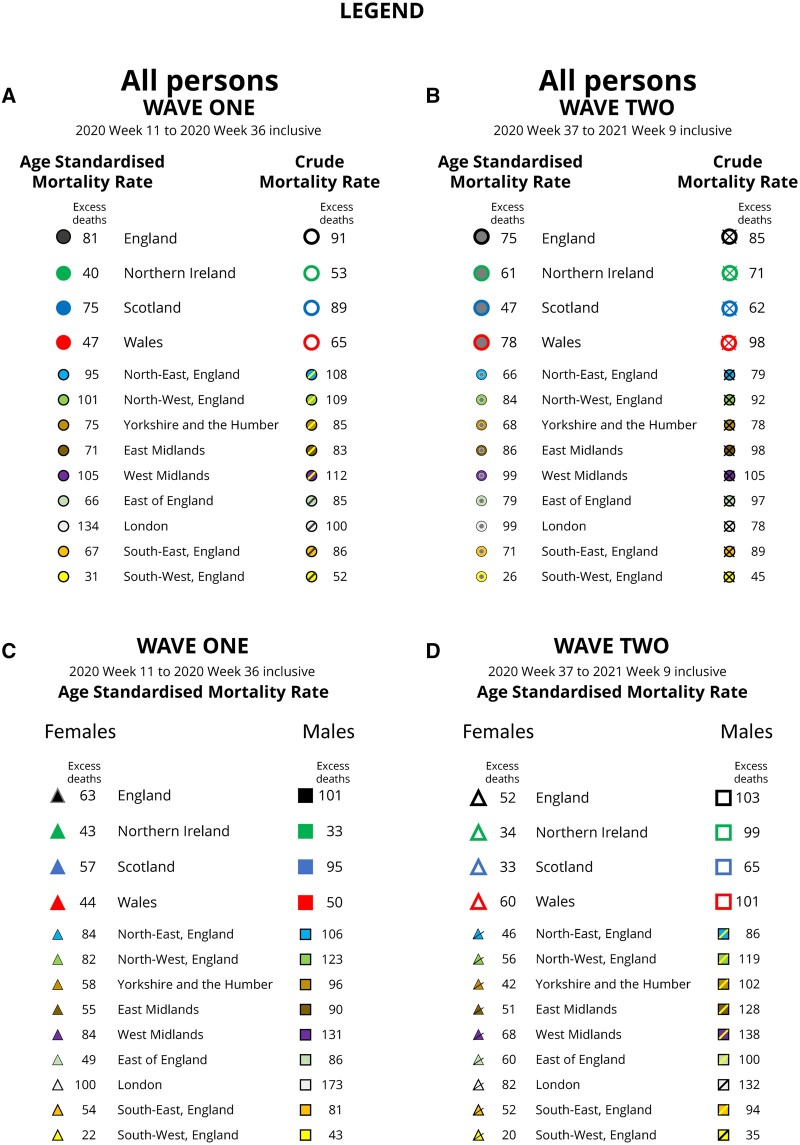
Expected and observed mortality rates by wave, country, region of England and sex.

In wave one, which covers the spring and summer, the expected crude mortality rate (CMR-sm) ranged from 265 per 100 000 in the London region to 504 in Wales (figure 1A). The expected ASMR-sm in wave one in London was 405 per 100 000 and Wales was 490. Thus, a relatively young London population (12% aged 65 years and older) accounts for much of the difference in expected crude mortality compared to Wales (21% aged 65 years and older).^5^ In wave two, which covers autumn and winter, the expected CMR-sm and ASMR-sm are around 11% higher (figure 1B). When age standardized, Scotland had the highest expected mortality rate (figure 1).

Similar levels of excess deaths were seen overall in both waves. The excess ASMR-sm was 77 per 100 000 in wave one and 73 in wave two. The least affected country or region was the South-West of England and the most affected was London. All person, excess ASMR-sm in the South-West of England was 31 per 100 000 in wave one and 26 in wave two. All person, excess ASMR-sm in London was 134 per 100 000 in wave one and 99 in wave two.

As expected, estimates of excess ASMR-sm are lower than excess ASMR-proj (figure S1). Excess ASMR-proj was 93 per 100 000 in wave one and 103 in wave two, overall. The South-West of England was least affected with an excess ASMR-proj of 50 per 100 000 in wave one and 55 in wave two. London was most affected with an excess ASMR-proj of 160 in wave one and 137 in wave two.

Excess age-standardized deaths were notably higher for males than females (figure 1C-D). Overall, male excess ASMR-sm was 58% higher than females in wave one (excess ASMR-sm per 100,00 for males in wave one was 96 compared to 61 for females). By excess ASMR-proj, the rate of male deaths was 50% higher than female in wave one (excess ASMR-proj per 100 000 for males in wave one was 114 compared to 76 for females). In wave two, the effect was larger. Overall, male excess ASMR-sm was 97% higher than females in wave two (excess ASMR-sm for males was 100 per 100 000 compared to 51 for females) and male excess ASMR-proj was 69% higher (excess ASMR-proj for males was 133 per 100 000 compared to 79 for females).

To facilitate comparison, relative absolute differences, and proportional differences, in excess ASMR were calculated using the South-West of England as the reference and used for ranking (figure S1). Apart from absolute excess being greater by the projected methodology, the ranking of countries and regions are broadly similar.

The largest increases in excess ASMR between waves was seen for males in Northern Ireland and Wales. Male excess ASMR-sm increased three-fold (excess ASMR-proj increased 2.5-fold) in wave two compared to wave one in Northern Ireland. In Wales the male excess ASMR-sm increased two-fold (excess ASMR-proj increased 1.9-fold). Most areas saw a decrease in female excess ASMR-sm in wave two, the largest fall in the North-East of England. The exceptions were Wales and the East of England. Wales had the largest increase in both female excess ASMR in wave two compared to wave one by either methodology (Supplementary figure S1).

## Discussion

The impact of COVID-19 on mortality is best measured by excess all cause deaths over that expected because it captures indirect consequences and deaths directly caused by COVID-19 but not directly attributed to it.^1–3^^,^^7^ Previous studies showed that estimates of expected deaths are sensitive to the method used.^7^ We applied a simple 5-year average as it is consistent and comparable with other outputs from government and have also used a projected trend for additional rigour. We are currently working across government and the devolved administrations to develop an agreed approach for the future outputs. To our knowledge, this is the first study of excess ASMR by sex, UK country, and region of England, across the first two waves of the COVID-19 pandemic in the UK. Previous studies have largely concentrated on crude excess death rates and not covered all regions or countries of the UK.^4–5^^,^^9^^,^^10^ Excess mortality is the difference between observed and expected deaths and can be expressed as an absolute rate or as a proportion. We have presented the data directly as scatterplots that allow the reader to assess the impact of expected rates based on a simple average to the observed rates. Absolute excess ASMRs produced by simple average and projected trend methods are provided in the Supplementary material. The comparisons reported here are broadly consistent using either method. Excess ASMRs were clearly greatest in London in wave one, consistently, irrespective of method. In wave two, there were more marginal differences between London and the West Midlands, with method impacting absolute rank.

## Supplementary Material

ckad144_Supplementary_Data

## Data Availability

Data used in this manuscript are publicly available on the relevant websites: England and Wales: https://www.ons.gov.uk/peoplepopulationandcommunity/birthsdeathsandmarriages/deaths/datasets/weeklyprovisionalfiguresondeathsregisteredinenglandandwales; Northern Ireland: https://www.nisra.gov.uk/statistics/death-statistics/weekly-death-registrations-northern-ireland; Scotland: https://www.nrscotland.gov.uk/statistics-and-data/statistics/statistics-by-theme/vital-events/general-publications/weekly-deaths-registered-in-scotland. Further information and/or clarification may be obtained from the corresponding author.
