# Correction to: A comparison of excess deaths by UK country and region during the first year of the COVID-19 pandemic

**DOI:** 10.1093/eurpub/ckad226

**Published:** 2023-12-29

This is a correction to: Neil A Hopper, Annie Campbell, Cath Roberts, Julie Ramsay, Jos IJpelaar, Myer Glickman, Vahé Nafilyan, Nazrul Islam, A comparison of excess deaths by UK country and region during the first year of the COVID-19 pandemic, *European Journal of Public Health*, 2023, ckad144, https://doi.org/10.1093/eurpub/ckad144

In the originally published version of this manuscript, there were errors in the way the corresponding author information was given:

- In the HTML, author Neil A Hopper is tagged as the corresponding author, however Nazrul Islam’s email was given in Dr Hopper’s author card. Nazrul Islam is the corresponding author.
- In the HTML and PDF, author Nazrul Islam’s email address is given in the ‘Correspondence’ line.
- In HTML and PDF, the affiliation given in the ‘Correspondence’ section (*Faculty of Medicine, University of Southampton, Southampton, UK*) doesn't match any of the affiliations in the affiliation list.
- In the PDF, the name of the corresponding author is missing in the ‘Correspondence’ line, as is journal style.

This error has been corrected online.

