# Supplementary figures and images for: A comparison of excess deaths by UK country and region during the first year of the COVID-19 pandemic
Source: Eur J Public Health. 2023 Oct 19;34(2):411–4. doi: 10.1093/eurpub/ckad144 (PMC10990540; doi:10.1093/eurpub/ckad144)

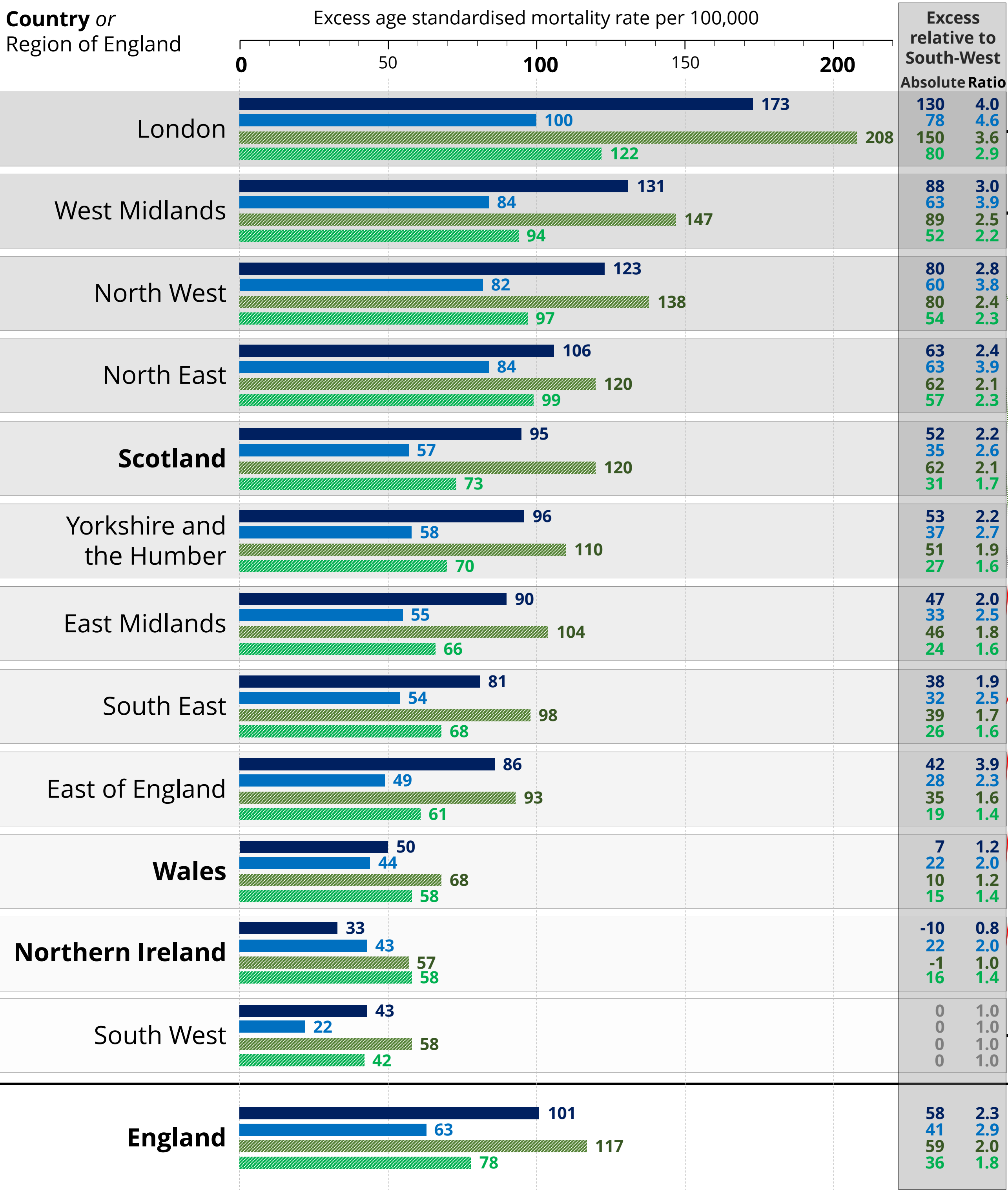

Wave one

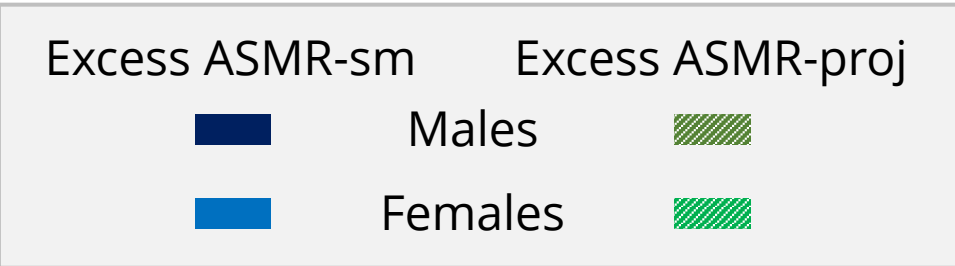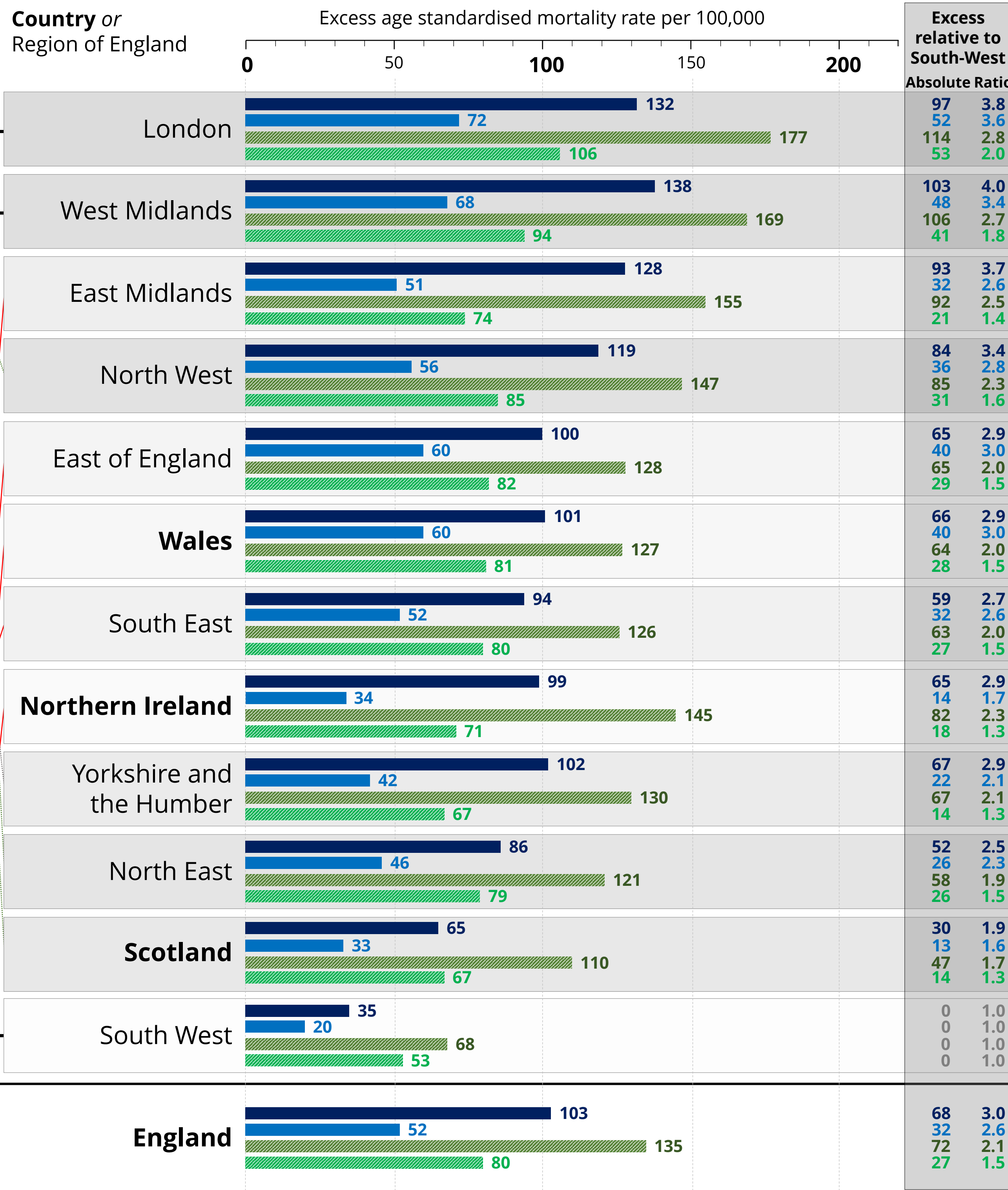

Wave two

Supplement: ckad144_Supplementary_Data [file ckad144_supplementary_data.pdf]
